# Twenty-four hour physical activity, sedentary behaviour and sleep profiles in adults living with rheumatoid arthritis: a cross-sectional latent class analysis

**DOI:** 10.1186/s44167-024-00049-5

**Published:** 2024-04-17

**Authors:** Lynne Feehan, Hui Xie, Na Lu, Linda C. Li

**Affiliations:** 1 Department of Physical Therapy, University of British Columbia, 212 Friedman Building, 2177 Wesbrook Mall, V6T 1Z3 Vancouver, BC Canada; 2 Faculty of Health Sciences, Simon Fraser University, 8888 University Drive, Burnaby, BC Canada; 3 Arthritis Research Canada, 230 - 2238 Yukon Street, Vancouver, BC Canada

**Keywords:** Rheumatoid arthritis, 24-hour movement, Sleep, Physical activity, Sedentary behaviour, Latent class analysis

## Abstract

**Background:**

Rheumatoid Arthritis (RA), an autoimmune systemic inflammatory disease, affects more than 17 million people globally. People with RA have higher risk of premature mortality; often experience chronic fatigue, pain and disrupted sleep; and are less physically active and more sedentary than healthy counterparts. It remains unclear how people with RA may balance sleep and awake movement activities over 24-hours, or how differences in 24-hour behaviours may be associated with determinants of health, or alignment with published activity guidelines.

**Methods:**

Cross-sectional exploration of objective measures of 24-hour sleep-wake activities in 203 people with RA. Latent Class Analysis (LCA) derived classes from time, by tertile, in six sleep-awake activities over 24 h. Comparisons of model fit statistics, class separation and interpretability defined best fit for number of classes. Variations in sleep-awake behaviour across classes and association of profile allocation with determinants of health, quality metrics for sleep, sitting and walking and alignment with published guidelines were explored. Multinomial logistic regression identified factors associated with likelihood of profile allocation.

**Results:**

LCA identified 2 to 6 classes and a 4-class model was determined as best fit for 24-hour sleep-awake behaviour profiles. One profile (26%) presented with more balanced 24-hour sleep, sitting and walking behaviours. The other three profiles demonstrated progressively less balanced 24-hour behaviours including: having low (< 7 h), high (> 8 h), or recommended (7–8 h) sleep duration in respective combination with high sitting (> 10 h), limited walking (< 3 h) or both when awake. Age, existing sitting and walking habit strength and fatigue were associated with likelihood of belonging to different profiles. More balanced 24-hour behaviour was aligned with better quality metrics for sleep, sitting and walking and published guidelines.

**Discussion:**

For people living with RA it is important to understand the ‘whole person’ and their ‘whole day’ to define who may benefit from support to modify 24-hour sleep-awake behaviours and which behaviours to modify. Supports should be informed by an understanding of personal or health-related factors that could act as barriers or facilitators for behavioural change, including exploring existing habitual sitting and walking behaviours.

**Trial registrations:**

ClinicalTrials.gov ID: NCT02554474 (2015-09-16) and ClinicalTrials.gov ID: NCT03404245 (2018-01-11)

**Supplementary Information:**

The online version contains supplementary material available at 10.1186/s44167-024-00049-5.

## Background

Rheumatoid Arthritis (RA) is the most common form of autoimmune systemic inflammatory joint disease. Rheumatoid Arthritis can present at any age, with onset commonly occurring between the ages of 30 and 60 [1]. It is estimated that in 2020, more than 17 million people were living with RA globally, with the prevalence varying from 50 to 200/100,000 people, and females 2 to 3 times more likely than males to have RA [2, 3]. Rheumatoid Arthritis typically presents with a rapid onset of pain, swelling and stiffness in the small joints of the hands and feet, as well as in multiple other joints. It commonly occurs on both sides of the body and does not settle over several weeks. Once diagnosed, treatment is primarily through medications with most people taking medications for the remainder of their life to reduce the number of joints involved and limit any damage to other organs, such as the heart, lungs and eyes [4].

People living with RA commonly have other chronic health conditions, including cardiovascular and respiratory conditions, diabetes, and depression [5, 6]. As such, adults living with RA have a markedly higher risk of progressive functional decline, reduced quality of life, and premature mortality [7]. People with RA often experience chronic sleep disruption, fatigue and pain, all of which may contribute to, or be exacerbated by, a reduced ability to participate in higher intensity activities and for being more sedentary throughout their day [8–10]. They are also less physically active and more sedentary than healthy people of similar age and sex [8, 11–13]. Reducing sedentary time, increasing light activity and higher intensity activities are independently associated with a greater likelihood of improved long-term health outcomes in this population [14, 15], similar to adults living with other chronic health conditions [16]. It remains unclear how people with RA may balance their time in sleep, sedentary activities and physical activity over 24 h [17, 18]. Additionally, little is known about how different combinations of objectively measured 24-hour sleep-movement patterns may be associated with meeting published evidence-based benchmarks for walking and Moderate to Vigorous Physical Activity (MVPA) or alignment with recommendations within 24-hour movement guidelines. It is also unknown how variations in 24-hour sleep-awake movement behaviors may be associated with personal demographic, socioeconomic, physical/mental health and other lifestyle characteristics associated with variations in sleep and physical activity in people with RA [19–21],.

In a previous exploratory study, we reported distinctly different patterns of 24-hour sleep-awake movement behaviors in 172 people living with osteoarthritis or inflammatory arthritis [22]. Notably, the study identified a subgroup of individuals who achieved a balance of sleep, moving and sitting throughout their day [22]. Our aim was to build on this previous work and focus specifically on a larger cohort of people living with RA. The objectives were to identify unique behaviour profiles of objectively measured sleep, nonambulatory activity, and walking behaviors over 24-hours and describe differences across the profiles for variations in time spent in different sleep and awake movement behaviors. We also wanted to explore differences across the profiles for alignment with selected quality metrics of sleep, sitting and walking and for meeting published evidence-based benchmarks for adults for daily steps [23], weekly MVPA [24], and selected elements within the Canadian 24-hour movement guidelines for adults [25, 26]. Finally, we wanted to examine the association between personal demographic, socioeconomic, physical and mental health, and walking and sitting habit characteristics and the likelihood of individuals living with RA belonging to different 24-hour sleep-awake movement behaviour profiles [27, 28].

## Methods

### Design

We conducted a cross-sectional analysis of baseline data from a cohort of 203 participants from two randomized clinical trials. Baseline assessments were completed between 2017 and 2022.

### Participants

The sample included people living with RA who consented to participate in one of two randomized clinical trials examining the efficacy of community-based, physical therapist lead, technology-enabled, physical activity counselling interventions (Online Physical Activity Monitoring in Inflammatory Arthritis (OPAM-IA) OR or On-demand Program to EmpoweR Active Self-management (OPERAS). ClinicalTrials.gov IDs: NCT02554474 (2015-09-16) and NCT03404245 (2018-01-11) [29, 30]. Baseline data were collected in both studies prior to randomization. Participants were recruited to either study from primarily urban rheumatology clinics in British Columbia (BC), Canada, through arthritis patient group networks or from postings on social media and Arthritis Research Canada’s website. There were no marked differences in eligibility criteria across the two studies. Individuals were eligible for both studies if they: (1) had a rheumatologist-confirmed diagnosis of RA [31], (2) had no surgery or injury to any joints in the previous 6 months, (3) had an email address and access to a computer or mobile device, (4) were able to participate in physical activities without health professional supervision, and (5) were able to speak and understand English [29, 30]..Further details of the specific eligibility criteria for each study can be found in the related publications [29.30].

### 24-hour sleep-awake activity measurement

Twenty-four hour sleep-awake activity was measured by research grade SenseWear MiniTM activity monitors (BodyMedia, Inc., Pittsburgh, PA). Sensewear (SW) monitors are multisensor devices that integrate personal demographic, physiologic and tri-axial accelerometry data. Sensewear monitors provide reliable and valid estimates of activity in people living with arthritis if worn for 4 or more days [32–34]. These devices have an excellent ability to distinguish between sedentary and light intensity activities (Positive Predictive Value: 0.81) [35]. They have also demonstrated high wake/sleep agreement (80%) and high sensitivity for sleep estimation (89%) compared to polysomnography and provide equivalent measures for time in bed compared to sleep diaries in free-living conditions [36, 37]. Sensewear monitors turn off when not in contact with skin, providing accurate measures of time off-body. Participants wore the monitors for 1 week on the upper arm over the triceps on the nondominant arm, and were instructed to only removed the device for showering or water-based activities.

### 24-hour sleep-awake activity data processing

Downloaded data from the devices were processed using Sensewear professional software (v8.1.0.22). The SW software uses proprietary algorithms to code each of the time-stamped minutes within each day (1440 min) for a number of characteristics, including the following used in this study: (1) sensor off-body, (2) lying down, (3) sleeping, (4) number of steps, and (5) Metabolic Equivalents (METs). The downloaded SW data were exported and further processed using MATLAB software (R2016a, The MathWorks, Inc., Natick, Massachusetts, United States). The daily minute-by-minute data were further coded in MATLAB into six discrete 24-hour sleep-awake activity categories, including time: (1) off-body (unknown activity, likely shower or bathing), (2) lying down sleeping, (3) lying down awake (resting), (4) awake in non-ambulatory activity (likely sitting, possibly standing still), (5) awake in intermittent ambulation (lower cadence walking), and (6) awake in purposeful ambulation (higher cadence walking). We used a 50-steps / minute cut-point to define intermittent (lower cadence) verses purposeful (higher cadence) walking [38]. Each minute within a day (1440 min) could only be classified into one of these six discrete behaviours over 24-hours.

In addition, we calculated total time in bed (sum of lying down sleeping + resting) and total walking time (sum of intermittent + purposeful walking). Finally, we also extracted time spent each day in bouted non-ambulatory activity (20 + minutes of uninterrupted non-ambulatory minutes, @ <1.5 Metabolic Equivalents (METs)) and MVPA (4 + METs), as well as, total daily steps. The final data used for each participant included the average value for the first four to six days, with at least 20 h of wear [32–34, 39]. From these data we calculated selected quality metrics for sleeping, sitting and walking behaviours. These included sleep efficiency (percentage of time sleeping while lying in bed), prolonged sitting behaviour (percentage of non-ambulatory time spent in bouted sitting), awake movement balance (percentage of time walking when awake), and walking efficiency (percentage of walking time spent in higher cadence ambulation).

### Self-reported personal demographic, socioeconomic, physical mental health and sitting walking habit data collection

Participants used online questionnaires to provide information on their personal demographic (age, sex, height, weight), socioeconomic [usual occupation (a person’s main occupation that is paid or unpaid work that takes most of their time and energy), highest education, annual household income, marital status], physical / mental health (pain, fatigue, depression), usual occupational and leisure time sitting habit strength and walking outside habit strength. Pain was measured with the short-form McGill Pain Questionnaire (SF-MPQ) using 15 pain-related words that can be rated from 0 (none) to 3 (severe). Scores vary from 0 to 45, with scores less than 15 indicating no to mildly discomforting levels of pain [40]. Fatigue was measured using the Fatigue Severity Scale (FSS), a nine-item questionnaire about fatigue and how it affects daily activities rated on a 7-point Likert scale (strongly disagree to strongly agree). A score of 4 or higher is considered to indicate clinically relevant fatigue [41, 42].. Depression was measured using the Patient Health Questionnaire-9 (PHQ-9), a nine-item questionnaire about common symptoms of depression rated on a 4-point frequency scale (1-not at all, 4-almost daily). A score of 5 or less indicates no or minimal depression [43, 44]. Participants rated their strength of habit for sitting during leisure time at home, sitting during usual occupational activities, and walking outside for 10 min or more using the Self-Reported Habit Index (SRHI). The SRHI is a 12-item scale, that rates specific behaviours done within a defined setting or context on a 7-point Likert scale (strongly disagree to strongly agree). Higher scores indicate a stronger habitual behaviour that is done frequently, automatically, and without thinking about it [45, 46].

### Statistical analyses

All the statistical analyses were conducted using SAS v9.4 software (SAS Institute, Inc., North Carolina, USA). All participants had 4 to 6 days of SW data with at least 20 h of wear. In addition, all participants provided complete baseline data for self-reported personal demographic, socioeconomic, physical / mental health and sitting / walking habit strength characteristics. There were no apparent differences in any self-reported baseline characteristic from either study, so these data were combined into one cohort for analyses (See Additional File 1. Supplementary Table 1: Baseline Characteristic Comparison: Whole cohort vs. study).

### Latent class analyses and model fit comparisons

We conducted a Latent Class Analysis (LCA) using time (minutes) spent in each of six discrete sleep-awake activity categories across 1440 min (24 h) [47]. We used the PROC LCA procedure in SAS [48]. For the LCA we categorized minutes in each of the six sleep-awake activities (continuous data) into lowest, middle and highest tertiles (ordinal data) to derive latent classes [49]. To determine the number of classes, we considered comparisons of model fit statistics [Akaike’s and Bayesian Information Criterion (AIC/BIC)], separation and interpretability of classes (across-class posterior probabilities for class allocation and across-class item probabilities by lowest, middle and highest tertiles) [50].

### 24-hour sleep-awake movement behaviour and quality metric comparisons

We compared differences across profiles and relative to the whole cohort for time spent in each of the six 24-hour sleep-awake activity categories. In addition, we compared differences for number of daily steps acquired and time spent in bed, prolonged sitting, walking at any cadence and MVPA. Finally, we compared differences across the profiles and relative to the whole cohort for quality metrics of sleeping, sitting and walking. We used descriptive statistics [mean and standard deviation (SD)] for these comparisons.

### Meeting evidence-based activity benchmarks

Using these descriptive comparisons, we identified the likelihood of individuals in the cohort and within each profile for meeting published evidence-based benchmarks for adults for daily step volume [6000 to 8000 steps/day] [23], and weekly MVPA volume (> 150 min/week) [24]. We also defined likely alignment with recommendations for sleep, sitting and higher intensity activity within the Canadian 24-hour sleep movement guidelines for adults (sleep: 7 to 8 h sitting: <10 h and MVPA: 25 + minutes) 25]. We used a 10-hour vs. 8-hour / day cut-point for objective measures of 24-hour non-ambulatory time, as described by Clarke et al. (2021) [26].

### Baseline characteristic comparisons

We compared differences across the identified profiles relative to the whole cohort for personal demographic, socioeconomic, physical/mental health, and sitting and walking habit strength. We also compared differences across profiles, relative to the whole cohort, for study participation and COVID-19 activity restriction. We used mean and SD for continuous variables and number and percentage for categorical variables for descriptive comparisons.

### Baseline characteristics and likelihood of profile allocation

We conducted multinomial logistic regression with backward elimination to identify baseline factors associated with a greater or lesser likelihood of individuals belonging to a specific profile, relative to a reference profile. We included personal demographic (age, sex, BMI), socioeconomic (marital partner, annual income > $80K, university education, employed), physical/mental health (pain, fatigue, depression), and sitting/walking habit strength, as well as study participation (OPAM vs. OPERAS) and COVID-19 related activity restrictions, as factors in the model. The baseline characteristics were all considered a priori as potentially associated with variations in sleep and activity participation in people with RA [19–21]. In addition, study participation and potential COVID-19 activity restrictions were included as factors in the model, as they were both considered a priori to be external (temporal) factors that may have influenced variations in participants sleep and awake activity behaviours. The effect of factors remaining in the final model are reported as Odds Ratio (ORs) with Wald 95% Confidence Intervals (95% CIs) relative to a reference profile (OR:1.0).

## Results

### Cohort characteristics

See Table 1 for further details of the baseline characteristics for the whole cohort. The cohort included 203 individuals who were predominantly female (92%), older (Mean: 56 years, SD: 13 years) and had a mean BMI of 28 (SD: 7) kg/m^2^. 58% (*n* = 118) of the cohort were recruited for the OPERAS study, and 39% (*n* = 80) were assessed when varying levels of mandated COVID-19 activity limitations were in place in BC. Less than half of the cohort were employed (45%), had a university degree (46%) or had an annual household income greater than $80K (41%). Whereas, 65% of the cohort had a spouse or partner. Of the cohort, 60% reported having no or minimal depression (PHQ-9 score ≤ 5). On average, the cohort also reported having mild pain (SF-MPQ Mean: 12, SD: 9) and clinically relevant levels of fatigue (FSS Mean: 4.7, SD: 1.3). In addition, the cohort reported having neither strong nor weak habitual leisure-time sitting (SRHI: Mean: 4.7, SD: 1.3), usual occupational sitting (SRHI: Mean: 4.5, SD: 1.7) or walking outside (SRHI: Mean 4.3, SD: 1.7) behaviours.

**Table 1:** Baseline characteristics: whole cohort vs. profiles

	High Sit / Low Walk (Most Inactive)	High Sleep / Low Walk	Low Sleep / High Sit	Most Balanced	Whole Cohort
Number [n (%)]	30 (15%)	63 (31%)	57 (28%)	53 (26%)	203 (100%)
Personal Demographics					
Age Years [Mean (SD)]	61.8 (12.7)	52.3 (12.6)	60.7 (12.5)	52.7 (11.9)	56.2 (13.0)
Sex = Female [n (%)]	25 (83.3%)	58 (92.1%)	52 (91.2%)	51 (96.2%)	186 (91.6%)
BMI - kg/m2 [Mean (SD)]	28.4 (6.6)	28.2 (8.8)	26.6 (5.9)	26.9 (5.8)	27.5 (7.0)
Socio-Economic Characteristics					
Employed = Yes [n (%)]	9 (30%)	30 (47.6%)	27 (47.4%)	26 (49.1%)	92 (45.3%)
Spouse/Common Law Partner = Yes [n (%)]	17 (56.7%)	38 (60.3%)	38 (66.7%)	39 (73.6%)	132 (65.0%)
Annual Household Income [n (%)]					
$80 K or less	17 (56.6%)	29 (46.0%)	20 (35.1%)	23 (43.4%)	89 (43.9%)
Over $80k	9 (30.0%)	24 (38.1%)	27 (47.4%)	23 (43.4%)	83 (40.9%)
Unknown	4 (13.3%)	10 (15.9%)	10 (17.5%)	7 (13.2%)	31 (15.3%)
University Degree = Yes [n (%)]	12 (40.0%)	29 (46.0%)	24 (42.1%)	29 (54.7%)	94 (46.3%)
Physical/Mental Health					
Depression (PHQ-9): Mild to Severe (Score ≥ 5) [n (%)]	17 (56.7%)	45 (71.4%)	28 (49.1%)	32 (60.4%)	122 (60.1%)
Fatigue (FSS) [1 to 7, Higher = More Fatigue [Mean (SD)]	4.6 (1.2)	5.1 (1.3)	4.3 (1.3)	4.8 (1.3)	4.7 (1.3)
Pain (SF-MPQ) [0 to 45, Higher = More Pain.[Mean (SD)]	12.4 (8.9)	14.4 (10.3)	9.5 (7.6)	11.5 (8.9)	12.0 (9.2)
Habit Strength					
Sitting at home, leisure time (SRHI) [1 to 7, Higher = Stronger Habit, Mean (SD)]	5.2 (1.0)	4.8 (1.3)	4.6 (1.3)	4.2 (1.3)	4.7 (1.3)
Sitting during usual occupational activity (SRHI) [1 to 7, Higher = Stronger Habit, Mean (SD))]	4.8 (1.5)	4.7 (1.7)	4.9 (1.4)	3.8 (1.7)	4.5 (1.6)
Walking, outside, > 10 min (SRHI) [1 to 7, Higher = Stronger Habit, Mean (SD)]	3.7 (1.6)	4.0 (1.8)	4.6 (1.5)	4.7 (1.7)	4.3 (1.7)
External (Temporal) Factors					
Covid Activity Restrictions = Yes [n (%)]	10 (33.3%)	29 (46%)	21 (36.8%)	20 (37.7%)	80 (39.4%)
Study = OPERAS [n (%)]	15 (50%)	40 (63.5%)	33 (57.9%)	30 (56.6%)	118 (58.1%)

BMI - Body Mass Index. SF-MPQ: Short Form-McGill Pain Questionnaire. FSS: Fatigue Severity Scale. PHQ-9: Patient Health Questionnaire-9. SRHI: Self-Reported Habit Index. OPERAS: On-demand Program to EmpoweR Active Self-management

### Cohort 24-hour sleep-awake behaviours

Table 2 shows details for time spent by the cohort in the six 24-hour sleep-awake activity categories included in the LCA. Table 3 shows details for time spent by the cohort in additional (calculated) 24-hour sleep-awake behaviours, quality metrics of sleeping / sitting / walking daily steps and MVP, and likelihood of meeting published activity benchmarks / guidelines.

**Table 2:** Across-profile vs. whole cohort comparisons: time (minutes) spent in each of the six discrete 24-hour sleep-awake activity categories included in the latent class analysis

	High Sit / Low Walk (Inactive)	High Sleep / Low Walk	Low Sleep / High Sit	Most Balanced	Whole Cohort
Number [n (%) ]	30 (15%)	63 (31%)	57 (28%)	53 (26%)	203 (100%)
Six Discrete 24-Hour Sleep-Awake Activity Categories: Minutes / Day [Mean (SD)]					
Off body (unknown activity, likely showering / bathing)	20.7 (11.2)	24.9 (13.6)	29.7 (15.8)	28.5 (25.7)	26.6 (18.0)
Lying Down Sleeping	440.1 (55.8)	516.8 (66.1)	404.7 (54.9)	435.4 (76.7)	452.7 (78.6)
Lying Down Awake (Resting)	85.8 (47.7)	104.9 (23.6)	59.0 (18.0)	94.9 (49.2)	86.6 (47.5)
Awake Non-Ambulatory (sitting / standing still)	746.6 (49.7)	626.8 (64.9)	746.8 (61.2)	581.9 (77.7)	670.9 (101.1)
Awake Intermittent Ambulation (walking, lower cadence)	106.2 (36.6)	143.7 (36.7)	169.7 (44.7)	256.4 (50.5)	174.9 (67.6)
Awake Purposeful Ambulation (walking, higher cadence)	10.6 (10.8)	23.0 (15.1)	30.0 (15.9)	42.9 (23.1)	28.3 (20.2)

**Table 3:** Across-profile vs. whole cohort comparisons: additional 24-hour sleep-awake behaviours, quality metrics for sleeping / sitting / walking, daily steps / MVPA volume, and meeting activity benchmarks / guidelines

	High Sit / Low Walk (Inactive)	High Sleep / Low Walk	Low Sleep / High Sit	Most Balanced	Whole Cohort
Number [n (%) ]	30 (15%)	63 (31%)	57 (28%)	53 (26%)	203 (100%)
Additional (Calculated) 24-Hour Sleep-Awake Behaviours: Minutes / Day [Mean (SD)]					
Time in Bed (lying down sleeping & awake)	525.9 (48.4)	621.7 (82.5)	463.8 (55.2)	530.2 (80.4)	539.3 (93.3)
Awake Non-Ambulatory (sitting / standing still)– Bouted*	447.1 (156.2)	312.3 (100.0)	392.5 (119.1)	209.6 (90.7)	332.4 (144.3)
Awake Non-Ambulatory (sitting / standing still)– Not Bouted	299.5 (139.6)	314.4 (84.9)	354.3 (90.5)	372.3 (75.0)	338.5 (97.5)
Awake Ambulatory (walking - intermittent & purposeful)	116.8 (40.8)	166.7 (43.1)	199.8 (43.2)	299.4 (57.0)	203.3 (78.2)
Quality Metrics for Sleeping, Sitting and Walking: Percentage (%) [Mean (SD)]					
Sleep Efficiency (% time in bed sleeping)	83.8% (8.6%)	83.5% (6.8%)	87.1% (4.0%)	82.1% (9.2%)	84.2% (7.4%)
Prolonged Sitting Behaviour (% time sitting spent in bouts of 20 + minutes)	61.1% (17.9%)	49.4% (14.1%)	52.0% (13.5%)	35.3% (12.9%)	48.2% (16.5%)
Awake Movement Balance (% time awake walking)	13.0% (4.4%)	20.9% (4.9%)	21.1% (4.5%)	34.0% (6.0%)	23.2% (8.6%)
Walking Efficiency (% walking time in higher cadence walking)	8.8% (6.9%)	13.0% (7.8%)	15.9% (10.0%)	14.1% (6.8%)	13.5% (8.4%)
Daily Steps (#) and MVPA (Minutes): [Mean (SD)]					
**Steps	2723.0 (1720.8)	4553.5 (1840.7)	5473.9 (1694.9)	8507.5 (2524.4)	5649.6 (2773.8)
***MVPA	6.8 (12.8)	15.2 (19.4)	18.3 (21.3)	25.4 (27.7)	17.5 (22.3)
Meeting Evidence-Based Activity Benchmarks / Guidelines					
Daily Steps (6000 to 8000/day) [Yes/No]	No	No	No	Yes (exceeds)	No
Weekly MVPA (> 150 min/week of higher intensity activity) [Yes/No]	No	No	No	Yes	No
24-hour Sleep-Movement Guidelines (7 to 8 h sleep, < 10 h sitting, 25 + minutes MVPA [meeting 0,1,2, or 3 elements]	1 (sleep)	0	0	3 (sleep, sit, MVPA)	1 (sleep)

* Bouted = uninterrupted period of sitting of 20 + minutes. ** Steps - includes steps accumulated through any type of ambulation at any intensity. *** MVPA (Moderate to Vigorous Activity) - includes time spent in any type of higher intensity activity

Participants in the cohort wore the SW devices for an average of 5.9 (SD:0.4, min-max:4–6) days, with an average daily non-wear (unknown activity, likely showering or bathing) time of 27 (SD:18) minutes / day. On average, participants spent 453 (SD:79) minutes / day sleeping, 671 (SD:101) minutes / day in non-ambulatory activity, 175 (SD:68) minutes / day in intermittent walking, and 28 (SD:20) minutes/day in purposeful walking. On average, they accumulated 5,650 steps a day (SD:2,774) and 17 min / day in MVPA (SD:22). Individuals spent 84% of their time in bed sleeping and 77% of their awake time sitting or standing still. Approximately 48% of their non-ambulatory time was spent in prolonged sitting. Only 23% of their awake time included ambulatory activities with 14% of their total walking time spent in higher cadence walking. Other than having recommended sleep duration, the cohort did not meet 24-hour movement guidelines recommendations for objective measures of time spent sitting or in higher intensity activity. People in the cohort also did not meet published evidence-based benchmarks for recommended daily steps or weekly MVPA.

### Latent class analyses and model fit comparisons

We examined the model fit statistics for latent class models with 2 to 6 classes for tertiles of time spent in each of the six 24-hour sleep-awake activity categories (Additional File 1. Supplementary Table 2: Model Fit Comparisons). The AIC for best fit (smaller better) selected 6 classes while BIC best fit (smaller better) selected 2 classes, which is consistent with the literature that indicates a BIC approach tends to penalize model complexity more and so tends to select simpler models than AIC [51]. However, both the AIC and BIC approaches showed a divergence (flattening) at 4 classes (Additional File 1. Supplementary Fig. 1: Across-class AIC / BIC model fit comparison plot). Taking both model fit statistics into consideration, the 4-class model appeared to be overall closest to the best model selected by either AIC (6-classes) or BIC (2-classes). These findings also suggested that the 4-classes model may have provide an adequate fit for latent class structure while remaining parsimonious.

For further clarity we defined behaviour profile names for each of the 4 latent classes using 24-hour sleep-awake behaviour descriptors. Profile names included: Most Balanced, Low Sleep / High Sit, High Sleep / Low Walk and High / Sit Low Walk. As shown below, these profile names are also consistent with interpretations of the 4 latent classes using item probabilities.

The examination of classes and item probabilities also support the selection of 4 classes for their intuitive interpretability. To explore the separation of the 4 latent classes we examined across-class predicted probabilities to define the probability of the class model accurately predicting class membership. We identified on average a 97% posterior probability for being correctly allocated to High Sit / Low Walk, a 96% posterior probability for being correctly allocated to Most Balanced, a 94% posterior probability for being correctly allocated to High Sit / Low Walk, and an 83% posterior probability for being correctly allocated to Low Sleep / High Sit profile allocation (Additional File 1. Supplementary Table 3: Across-Class Predicted Probabilities: Four-class model fit).

In addition, we explored across-class item probabilities comparisons for each of the six 24-hour sleep-wake activities included in the LCA by the lowest, middle and highest tertile classifications. Findings from this analyses also support the interpretability of using a 4-class model fit. These findings show a consistent trend across-classes for those identified within the highest or lowest tertiles for specific sleep or awake behaviours showing a corresponding high (> 80%) or low (< 20%) probability of being allocated to a profile with a similar high or low sleep-awake behaviour characteristic. For example, those with the lowest higher cadence walking behaviours and the highest non-ambulatory behaviours showed a very high probability (> 90%) probability for being allocated to the High Sit / Low Walk (inactive) profile. Conversely, those with the lowest low cadence walking behaviour and the highest non-ambulatory behaviour showed a very low probability (< 10%) of being allocated to the Most Balanced profile. Additionally, those with the lowest or highest sleep duration had a very low probability (< 10%) for being allocated respectively to the High Sleep / Low Walk and Low Sleep / High Sit profiles. (Additional File 1. Supplementary Table 4: Across-class item probability by tertile classification (Lowest, Middle, Highest): Four-class model fit).

### Across-profile comparison: 24-hour sleep and awake activity behaviours

**Fig. 1:**
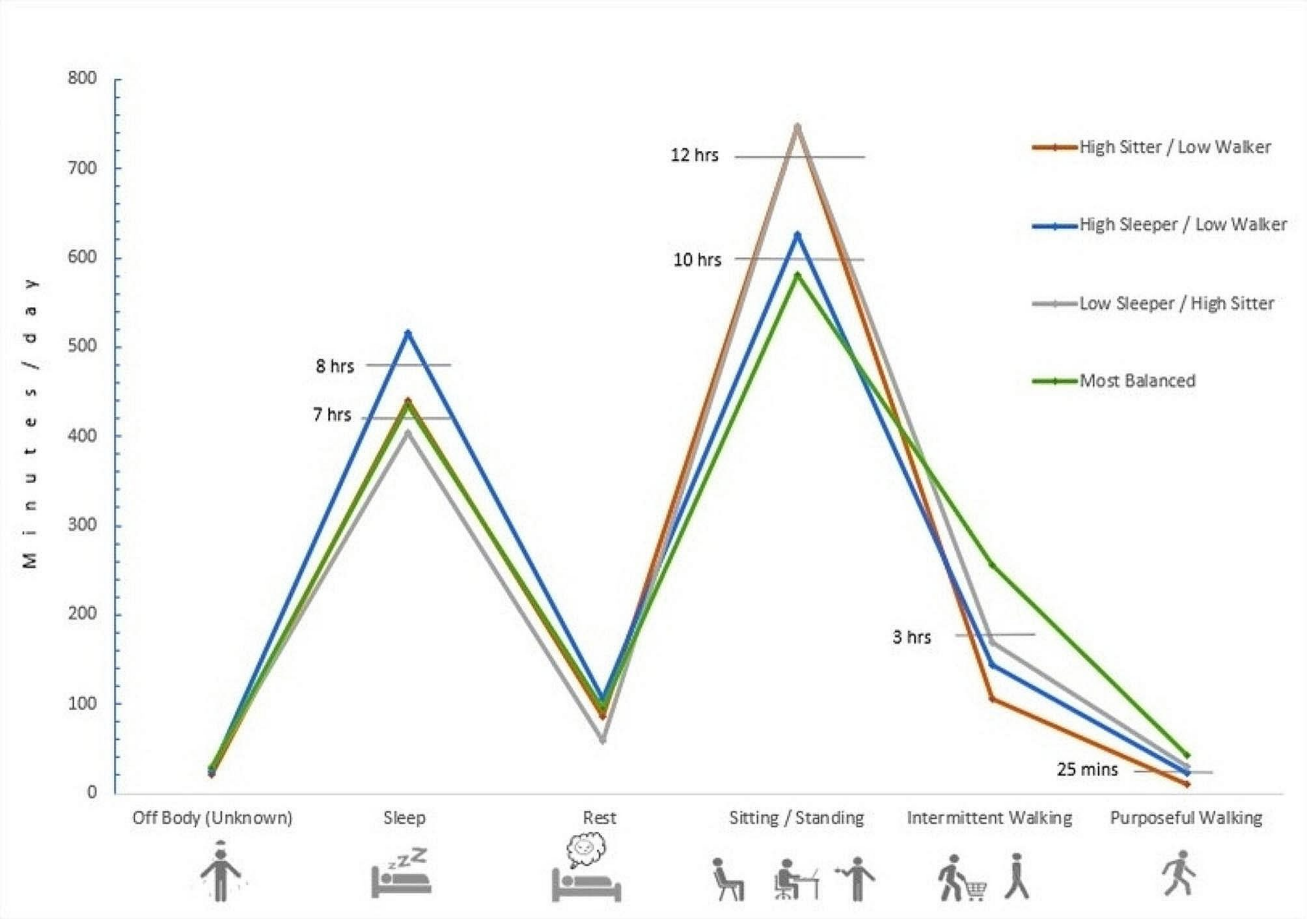
Across-profile comparisons: Average time spent in each of six discrete sleep-awake activity categories over 24-hours (1440 min) The images below the figure from left to right represent the following sleep-awake activities: (1) off-body time (unknown activity, likely showering/bathing), (2) lying down asleep, (3) lying down awake (resting), (4) awake non-ambulatory activities (likely sitting or standing still), (5) awake intermittent (lower cadence) walking, and (6) awake purposeful (higher cadence) walking. Average time in each sleep or awake activity category is identified in minutes / day (Y axis), with additional markers embedded in the figure to identify key daily cut- points for time spent sleeping (< 7 and > 8 h), sitting / standing still (< 10 and > 12 h), walking intermittently (lower cadence walking) (< 3 h) and walking purposefully (higher cadence walking) (< 25 min)

Figure 1 shows a comparison of time spent in each of the six sleep-awake activity categories across 24-hours for the 4 profiles identified. Table 2 also shows across-profile details for time spent in the six 24-hour sleep-awake activity categories included in the LCA. Table 3 also shows across-profile details for time spent in additional (calculated) 24-hour sleep-awake behaviours, daily steps and MVPA, and likelihood of meeting published activity benchmarks / guidelines. Overall, there were no notable differences in the duration of daily non-wear time across profiles. The mean off-body time (unknown activity, likely showering or bathing) across profiles varied from 21 to 30 min / day; accounting from 1.5 to 2% of the total 1440 minutes in a day [Table 2; Fig. 1].

We identified one profile of 53 individuals (26%) with an overall more balanced 24-hour sleep-awake activity pattern (*Most Balanced Profile*). Individuals in this profile averaged 435 (SD:77 min) minutes of sleep and 582 (SD:78) minutes in non-ambulatory activities. They also averaged 256 (SD:51) minutes of intermittent walking and 43 (SD:23) minutes of purposeful walking, accumulating an average of 8508 (SD: 524) steps a day. Those in this profile also averaged 25 (SD:28) minutes a day of higher intensity activity. As such, individuals in this more balanced profile were likely to meet recommended sleeping, sitting and MVPA criteria within the 24-hour sleep-movement guidelines. In addition, individuals in this profile exceeded published benchmarks for recommended daily steps and weekly MVPA [Tables 2 and 3 Fig. 1].

We identified a second smaller profile of 30 individuals (15%) in which, although sleeping 440 (SD:56) minutes a day on average, they demonstrated a more inactive lifestyle when they were awake (*High Sit / Low Walk Profile*). Individuals in this most inactive profile spent an average of 747 (SD:50) minutes a day engaged in non-ambulatory activities. In addition, they only averaged 106 (SD:37) minutes a day in intermittent walking and 11 (SD:11) minutes a day in purposeful walking, accumulating on average only 2723 (SD:1721) steps a day. Additionally, they averaged only 7 (SD:13) minutes a day in higher intensity activities. As such, members in this most inactive profile met only the sleep recommendations of the within the 24-hour movement guidelines and did not meet the daily steps or weekly MVPA benchmarks [Tables 2 and 3; Fig. 1].

We also identified two additional profiles characterized by either low (< 7 h) or high (> 8 h) sleep duration [52]. The participants in the profile with higher sleep duration *(High Sleep / Low Walk Profile, 31%)* averaged 517 (SD:66) minutes of sleep and 627 (SD:65) minutes of non-ambulatory activities. Individuals in this profile averaged 144 (SD:37) minutes a day in intermittent walking and 23, (SD:15) minutes a day in purposeful walking, accumulating an average of 4554 (SD: 1841) daily steps. Those in this profile also averaged 15 min a day (SD:19) in higher intensity activities. Therefore, individuals in this high sleep / low walk profile did not meet any of the recommended elements within the 24-hour movement guidelines or the daily step or weekly MVPA benchmarks [Tables 2 and 3; Fig. 1]. Participants in the profile with lower sleep duration *(Low Sleep / High Sit Profile, 28%)* averaged 517 (SD:66) minutes of sleep and 747 (SD:6 ) minutes in non-ambulatory activities. Individuals in this profile walked intermittently 170 min a day on average (SD:45) and purposefully for 30 (SD:16) minutes a day, accumulating a mean of 5474 (SD:1695) steps each day. In addition, they spent an average of 18(SD: 21) minutes a day in higher intensity activities. As such, individuals in this low sleep / high sit profile also did not meet any of the recommended 24-hour movement guideline criteria or the daily step or the weekly MVPA benchmarks [Tables 2 and 3; Fig. 1].

### Across-profile comparison: quality metrics of sleeping, sitting and walking

See Table 3 for further across-profile details of quality metrics of sleep, sitting and walking. All the profiles demonstrated an average sleep efficiency greater than 80% [53]. However, only the low sleep profile had a sleep efficiency greater than 85% (Mean: 87%, SD:4%) [50]. Indicating that although individuals in the low sleep profile spent less time in bed, they were the most efficient sleepers. Progressing from the most balanced through to the most inactive profile, the time spent in prolonged sitting behaviours progressively increased. With participants in the most balanced profile spending an average of 35% (SD:13%) of their non-ambulatory time in prolonged sitting activities. Whereas, those in the most inactive profile spent on average 61% (SD:18%) of their non-ambulatory time in prolonged sitting activities. This finding indicates that people in the most inactive profile were not only sitting for a greater percentage of time in their day, they also spent a greater percentage of sitting time in prolonged sitting activities. Conversely, when progressing from the most balanced through to the most inactive profiles, the time spent walking when awake progressively decreased. The most balanced profile spent 34% (SD:6%) of their time awake walking on average, while the most inactive profile spent 13% (SD:4%) of their awake time walking on average. Notably, all but the most inactive profile had similar walking efficiency, with the average proportion of walking time spent in higher cadence ambulation activities varying from 14 to16% in the more balanced, low sleeper and high sleeper profiles. Whereas, on average, those in the most inactive profile spent only 9% (SD:7%) of their walking time in higher cadence walking activities. This finding indicates that not only were people in the most inactive profile spending a lower percentage of their day walking, they also spent a smaller percentage of their walking time in higher cadence walking activities.

### Across-profile vs. cohort comparisons: baseline characteristics

Table 1 also shows across-profile details of baseline characteristics relative to the whole cohort. Relative to those in the whole cohort, those in the most balanced and high sleep profiles were younger compared to those in the low sleep and most inactive profiles being older. Relative to those in the whole cohort, those in the most balanced profile were more likely to have a spouse or partner, a university education and an annual household income greater than $80, with the opposite trend for difference in these same socioeconomic characteristics in the most inactive profile. Compared to those in the whole cohort, those in the low sleep profile reported lower levels of fatigue and pain. Whereas, those in the high sleep profile reported higher levels of fatigue and pain relative to the whole cohort. Those in the most balanced profile reported lower leisure time and usual occupational sitting habits and higher walking outside habit scores relative to the whole cohort. Which is in contrast to those in the most inactive profile, reporting higher leisure time and usual occupational sitting habits and lower walking outside habits relative to the whole cohort. Finally, there was no apparent difference across profiles for the proportion of those in either study or for those assessed during COVID-19 activity restrictions [Table 1].

### Baseline characteristics and likelihood of profile allocation

Table 4 shows the likelihood (OR; 95% CI)) of being allocated to different profiles relative to a reference profile for each baseline personal demographic, socioeconomic, health, and habit characteristic remaining in the model following multivariate regression analyses. The analysis findings highlight that the determinants for the likelihood of allocation to different of 24-hour sleep-movement behaviour profiles were multifactorial. Individuals were more likely to be allocated to the more balanced profile, than to the most inactive profile if they were younger (OR: 0.94, 95% CI: 0.90–0.98), had stronger walking outside habits (OR:1.44, 95% CI: 1.05–1.97) or had weaker leisure-time sitting habits (OR:0.62, 95% CI: 0.39–0.98 ). In addition, compared to the low sleep / high-sit profile, weaker usual occupational sitting habits was also associated with a greater likelihood of being in the more balanced profile (OR: 0.61, 95% CI: 0.45–0.81). Stronger walking outside habits was also associated with a greater likelihood of being in the low sleep / high sit profile than in the most inactive profile (OR: 1.36, 95% CI: 1.01–1.84). While, younger age (OR:0.94, 95% CI: 0.90–0.98) and greater fatigue (OR:1.59, 95% CI: 1.07–2.36) were associated with a greater likelihood of being allocated to the high sleep / low walk profile relative to the most inactive profile. The analyses also revealed that sex, BMI, socioeconomic factors, pain status, depression status, the study individuals volunteered for and the potential impacts of COVID-19 activity restrictions were not associated with the likelihood of profile allocation.

**Table 4:** Odds ratios (OR) and 95% confidence intervals for the likelihood of profile allocation relative to a reference profile (OR:1.0) for baseline characteristics remaining in the multivariate regression analysis model

		Most Balanced	Low Sleep / High Sit	High sleep / Low Walk	High Sit / Low Walk
Factors included in regression model	Factors remaining in regression model				
*Personal Demographic Characteristics*: Age (Years), Sex (F vs. M), BMI (kg/m2)	Age	**0.94 (0.90–0.98)**	0.99 (0.95–1.04)	**0.94 (0.90–0.98)**	1.0
*Socio-Economic Factors*: Spouse/ Partner (yes/no), University Education (yes/no), Annual Household Income (+/- $80K, unknown), Employed (yes/no)	None	n/a	n/a	n/a	n/a
*Physical/Mental Health Indicators*: Pain (score), Fatigue (score), Depression - Mild to Severe (yes/no)	Fatigue	1.47 (0.98–2.21)	0.97 (0.67–1.40)	**1.59 (1.07–2.36)**	1.0
*Sitting Habits*: Home Leisure, Usual Occupational (score)	Sitting - Home Leisure	**0.62 (0.39–0.98)**	0.65 (0.40–1.01)	0.72 (0.45–1.14)	1.0
	*Sitting - Usual Occupational	**0.61 (0.45–0.81)**	1.0	0.76 (0.57, 1.02)	0.81 (0.58, 1.13)
	Sitting - Usual Occupational	0.74 (0.54–1.03)	1.24 (0.88–1.73)	0.94 (0.68–1.29)	1.0
*Walking Habits*: Walking Outside > 10 min (score)	Walking - Outside	**1.44 (1.05–1.97)**	**1.36 (1.01–1.84)**	1.15 (0.85–1.55)	1.0
Study Participation: OPERAS (yes/no)	None	n/a	n/a	n/a	n/a
Covid Activity Restriction: (yes/no)	None	n/a	n/a	n/a	n/a

**Bold** = Significantly lower (OR: <1.0) or higher OR: (> 1.0) likelihood for profile allocation relative to the reference profile (OR: 1.0). * Most balanced profile vs. low sleep / high sit profile as reference

## Discussion

This study explored objectively measured sleep and awake behaviours in a cohort of people living with RA to identify unique patterns of 24-hour sleep and awake movement behaviours and their associations with common personal, socioeconomic, physical, mental and existing sitting and walking habits. This study also identified how different patterns of 24-hour behaviours were associated with variations in quality metrics of sleep, sitting and walking and the likelihood of meeting evidence-based benchmarks for steps, MVPA and alignment with 24-hour movement guidelines.

We found that the cohort as a whole had acceptable sleep duration and efficiency. However, they spent more than three quarters of their awake time sitting, with almost half of their sitting time comprised of prolonged sitting activities. In addition, not only were people in the cohort spending less than a quarter of their awake time walking they were also only spending a small portion of their walking time in higher cadence walking activities. As such, other than getting acceptable sleep, the cohort as a whole did not meet benchmarks for walking, higher intensity activity or balanced 24-hour movement behaviour. These findings are consistent with previously published studies showing that, on average, people with RA are generally more sedentary and less active than similarly aged people and commonly do not meet recommended daily steps or weekly MVPA recommendations [54].

When we used LCA to explore this further, we identified four distinctive patterns for how people living with RA spent time sleeping and in different awake movement activities throughout their day. One profile, representing a quarter of the cohort, presented with a more balanced 24-hour sleep-awake activity behaviour. Whereas, those in the other three profiles demonstrated progressively less balanced behaviour profiles: having either low (< 7 h), high (> 8 h), or adequate (7–8 h) sleep duration in respective combination with having higher levels of sitting (> 12 h), limited walking activity (< 3 h) or having both high levels of sitting and low levels of walking. We also found that having more balanced 24-hour sleep-movement behaviours was associated with better quality metrics for sleep, sitting and walking and a greater likelihood of meeting evidence-based benchmarks for daily steps, MVPA and alignment with recommended times for sleep, sitting and higher intensity activities outlined in the Canadian 24-hour movement guidelines. Together, these findings suggest that many people living with RA can have a more balanced 24-hour sleep-awake activity lifestyle, which may in turn be associated with better physical and mental health. Future research should explore how more balanced and various combinations of less balanced 24-hour sleep and awake movement behaviours may be associated with improved health outcomes in people living with RA and other chronic health conditions.

Our findings also highlight the importance of tailoring healthy lifestyle messages based on how individuals are actually spending their time sleeping, sitting and walking throughout their day. For some, the message would be that they are doing well in terms of balancing their time in sleep, sitting and walking behaviours throughout their day. In others, who have lower levels of sleep and spend many hours sitting, the focus should be on finding opportunities to replace sitting at home or during their usual occupational activities with more time in bed sleeping (*sleep more / sit less*). Whereas, those with higher levels of sleep in combination with low levels of walking outside their home, the focus would be more on finding opportunities to replace time in bed with outdoor walking activities (*walk more / sleep less*). Alternately, for others who have acceptable sleep but are inactive when awake, then the attention would be on finding ways to replace sitting with walking activities (*walk more / sit less*) [18, 28].

A distinctive finding is the association between existing sitting and walking habits and the likelihood of having a more or less balanced 24-hour sleep-activity profile while living with RA. Habitual behaviours are actions, or series of actions, that occur with limited conscious thought, often in response to contextual or environmental cues [55, 56]. The relationship between existing habits and future behaviours is complex, as habits can have both moderating and mediating effects on future behaviours [57, 58]. Pre-existing habits can be a predictor of future behaviours, independent of the intention to perform a behaviour (i.e., habit as a mediator of future behaviour) [59]. As such, strong existing habitual behaviours are likely to increase the likelihood of future similar behaviours in similar contexts. However, strong existing habitual behaviours can also moderate the potential effect of the intention or desire to change behaviours in similar contexts [60]. This speaks to the old adage that old habits may be hard to break, which in turn may explain in part why strategies supporting someone to be more physically active do not necessarily change their existing sitting behaviours [61]. These findings support further explorations of the influence of strong or weak sitting or walking habits on activity-related health behaviour change interventions in future investigations [56].

Our findings also highlight that differences in age and physical or mental health may also be associated with having more or less balanced 24-hour sleep and movement profiles. Notably, age is not a modifiable factor. However, our findings support the value of understanding not only the potential influence of existing habitual behaviours but also the importance of managing co-existing factors like fatigue and pain when supporting a persons’ capacity, opportunity or motivation to modify their daily sleeping or movement behaviours [54, 62].

This study has limitations. Our findings have limited generalizability to people living with other chronic health conditions or people living with RA who are not inclined to volunteer for research studies or those with RA who did not meet the eligibility criteria for either study. This was an exploratory, cross sectional study, so any associations between differences in 24-hour sleep-awake activity behaviours and baseline characteristics could not be defined in terms of the directionality of these relationships. It is possible that living with RA affects a person’s 24-hour sleep and movement activity behaviours and/or that variations in 24-hour sleep-activity behaviours impact physical, mental or other health outcomes in people living with RA [63]. Future studies should explore these potential relationships using longitudinal observational or experimental study designs. Another limitation is our use of a research grade activity tracker to objectively measure 24-hour sleep and movement patterns, as these devices are expensive and not readily available. However, in clinical or usual life situations where accurate minute-by-minute data for research purposes are not needed, it is reasonable to consider using more affordable, accessible and acceptable consumer wearable activity trackers [64, 65]. Consumer wearable devices can provide reasonable objective estimates of patterns of time spent in different activities over 24 h to help guide and inform strategies to support individuals to monitor and modify their 24-hour sleep and movement behaviours [66].

## Conclusion

For people living with RA it is important to understand the ‘whole person’ and their ‘whole day’ to help define who may benefit from support with modifying their 24-hour sleep-movement behaviours. The findings also highlight the importance of tailoring healthy lifestyle messages based on how individuals are actually spending their time sleeping, sitting and walking throughout their day. Ideally, the planning and implementation of supports to modify behaviours should be guided by objective measures of sleep and awake activities and a shared-decision-making approach should be adopted to ensure that personal preferences and priorities are considered [21, 54, 59, 67]. In addition, supports should be informed by an understanding of potentially modifiable personal or health-related factors that could act as barriers or facilitators to behavioural change [68–70]. Including, exploring existing sitting or walking habitual behaviours, so that positive habits can be reinforced and strategies can be defined to help people identify and modify less positive habitual behaviours.

## Electronic supplementary material

Below is the link to the electronic supplementary material.

Supplementary Material 1

## Data Availability

Data are available from the corresponding author with a reasonable request.

## Abbreviations

95% CI: 95% Confidence Intervals
AIC / BIC: Akaike’s and Bayesian Information Criterion
FSS: Fatigue Severity Scale
LCA: Latent Class Analysis
METs: Metabolic Equivalents
MVPA: Moderate to Vigorous Physical Activity
OPAM-IA: Online Physical Activity Monitoring in Inflammatory Arthritis
OPERAS: On-demand Program to EmpoweR Active Self-management
OR: Odds Ratio
PHQ-9: Patient Health Questionnaire-9
RA: Rheumatoid Arthritis
SD: Standard Deviation
SF-MPQ: Short Form-McGill Pain Questionnaire
SRHI: Self-Reported Habit Index
