# Supplementary material for: Twenty-four hour physical activity, sedentary behaviour and sleep profiles in adults living with rheumatoid arthritis: a cross-sectional latent class analysis
Source: J Act Sedentary Sleep Behav. 2024 Apr 17;3:10. doi: 10.1186/s44167-024-00049-5 (PMC11960349; doi:10.1186/s44167-024-00049-5)
Supplement: Supplementary file 1 — Supplementary Material 1 [file 44167_2024_49_MOESM1_ESM.pdf]

**Supplementary Table 1**: Baseline Personal Demographic, Socioeconomic, Physical / Mental Health and Sitting / Waling Habit Characteristics: Whole Cohort vs Study			
	Whole Cohort	OPAM-IA	OPERAS
Number [n (%)]	203 (100%)	85 (100%)	118 (100%)
Personal Demographics			
Age Years [Mean (SD)]	56.2 (13.0)	56.9 (13.4)	55.7 (12.8)
Sex = Female [n (%)]	186 (91.6%)	76 (89.4%)	110 (93.2%)
BMI - kg/m2 [Mean (SD)]	27.5 (7.0)	28.2 (8.1)	26.9 (6.1)
Socio-Economic Characteristics			
Employed = Yes [n (%)]	92 (45.3%)	36 (42.4%)	56 (47.5%)
Spouse/Common Law Partner = Yes [n (%)]	132 (65.0%)	52 (61.2%)	80 (67.8%)
Annual Household Income [n (%)]			
$80 K or less	89 (43.9%)	44 (51.8%)	45 (38.4%)
Over $80k	83 (40.9%)	27 (31.8%)	56 (47.5%)
Unknown	31 (15.3%)	14 (16.5%)	17 (14.4%)
University Degree = Yes [n (%)]	94 (46.3%)	41 (48.2%)	53 (44.9%)
Physical/Mental Health			
Depression (PHQ-9): Mild to Severe (Score ≥5) [n (%)]	122 (60.1%)	50 (58.8%)	72 (61.0%)
Fatigue (FSS) [1 to 7, Higher = More Fatigue [Mean (SD)]	4.7 (1.3)	4.8 (1.4)	4.6 (1.3)
Pain (SF-MPQ) [0 to 45, Higher = More Pain.[Mean (SD)]	12.0 (9.2)	13.8 (10.5)	10.7 (7.9)
Habit Strength			
Sitting at home, leisure time (SRHI) [1 to 7, Higher = Stronger Habit, Mean (SD)]	4.7 (1.3)	4.8 (1.2)	4.5 (1.3)
Sitting during usual occupational activity (SRHI) [1 to 7, Higher = Stronger Habit, Mean (SD))]	4.5 (1.6)	4.4 (1.7)	4.6 (1.6)
Walking, outside, > 10 minutes (SRHI) [1 to 7, Higher = Stronger Habit ,Mean (SD)]	4.3 (1.7)	4.0 (1.6)	4.5 (1.7)
BMI - Body Mass Index. SF-MPQ: Short Form-McGill Pain Questionnaire. FSS: Fatigue Severity Scale. PHQ-9: Patient Health Questionnaire-9. SRHI: Self-Reported Habit Index. OPERAS: On-demand Program to EmpoweR Active Self-management. OPAM-IA: Online Physical Activity Monitoring in Inflammatory Arthritis			

**Supplementary Table 2:** Model Fit Comparisons						
Class #	G-squared	AIC	BIC	CAIC	Adjusted BIC	Entropy
Whole Cohort	678.11	702.11	741.87	753.87	703.85	1
Two	577.93	627.93	710.76	735.76	631.55	1
Three	520.77	596.77	722.67	760.67	602.27	0.91
Four	470.27	572.27	741.24	792.24	579.66	0.89
Five	429.04	557.04	769.08	833.08	566.31	0.89
Six	398.7	552.7	807.82	884.82	563.86	0.89

Supplementary Figure 1: Across-Class AIC vs BIC Comparisons

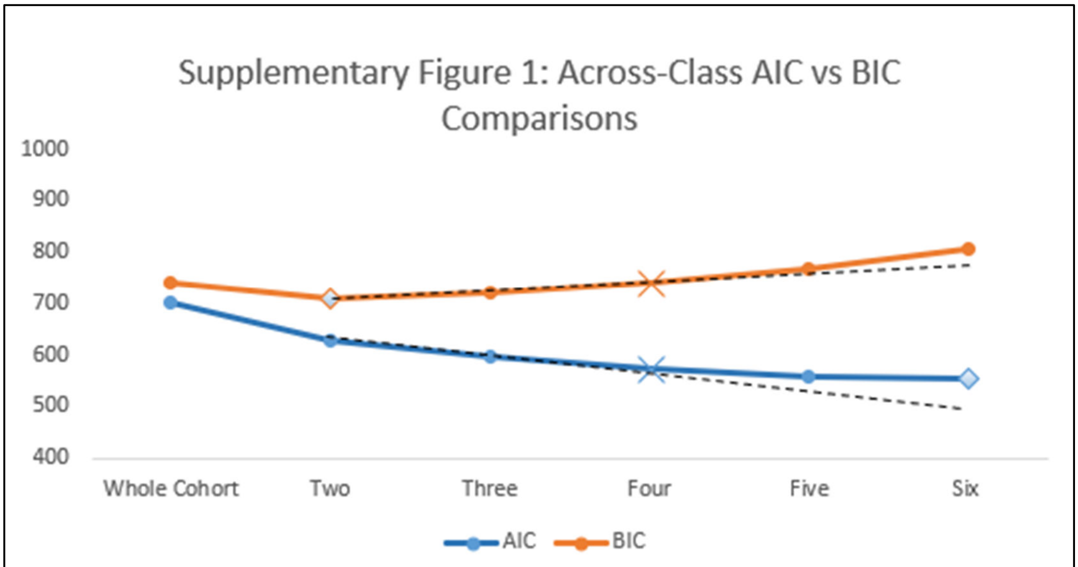

Supplementary Table 3: Across-Class Predicted Probabilities: Four-class model fit

Profile Name	CLASS #	N Obs	Label	N	Mean	Std Dev
High Sleep / Low Walk	**1**	63	**Latent Class 1 Posterior Probability**	63	**0.97**	0.07
			Latent Class 2 Posterior Probability	63	0.01	0.02
			Latent Class 3 Posterior Probability	63	0.00	0.00
			Latent Class 4 Posterior Probability	63	0.02	0.07
High Sit / Low Walk (Inactive)	**2**	30	Latent Class 1 Posterior Probability	30	0.00	0.00
			**Latent Class 2 Posterior Probability**	30	**0.94**	0.12
			Latent Class 3 Posterior Probability	30	0.00	0.00
			Latent Class 4 Posterior Probability	30	0.06	0.12
Most Balanced	**3**	53	Latent Class 1 Posterior Probability	53	0.00	0.00
			Latent Class 2 Posterior Probability	53	0.00	0.00
			**Latent Class 3 Posterior Probability**	53	**0.96**	0.11
			Latent Class 4 Posterior Probability	53	0.04	0.11
Low Sleep / High Sit	**4**	57	Latent Class 1 Posterior Probability	57	0.05	0.12
			Latent Class 2 Posterior Probability	57	0.07	0.13
			Latent Class 3 Posterior Probability	57	0.06	0.15
			**Latent Class 4 Posterior Probability**	57	**0.83**	0.19

BOLD: Probability of assignment to class of same number

**Supplementary Table 4**: Across-Class Item Probability by Tertile Classification (Lowest, Middle, Highest): Four-class model fit				
Profile	High Sit / Low Walk (Inactive)	High Sleep / Low Walk	Low Sleep / High Sit	Most Balanced
Number (%)	30 (15%)	63 (31%)	57 (28%)	53 (26%)
Lowest Tertile (time in each activity is low)				
Off body (unknown activity, likely showering / bathing)	0.40	0.34	0.26	0.34
Lying Down Sleeping	0.44	*0.00*	0.59	0.40
Lying Down Awake (resting)	0.46	*0.17*	0.51	0.27
Awake Non-Ambulatory (sitting / standing still)	*0.00*	0.42	*0.00*	0.74
Awake Intermittent Ambulation (walking, lower cadence)	**0.84**	0.44	0.22	*0.00*
Awake Purposeful Ambulation (walking, higher cadence)	**0.90**	0.38	*0.16*	*0.10*
Middle Tertile (time in each activity is moderate)				
Off body (unknown activity, likely showering / bathing)	0.35	0.27	0.41	0.33
Lying Down Sleeping	0.31	0.33	0.41	0.29
Lying Down Awake (resting)	*0.13*	0.37	0.49	0.26
Awake Non-Ambulatory (sitting / standing still)	*0.04*	0.58	0.30	0.26
Awake Intermittent Ambulation (walking, lower cadence)	*0.16*	0.56	0.53	*0.00*
Awake Purposeful Ambulation (walking, higher cadence)	*0.00*	0.37	0.47	0.36
Highest Tertile (time in each activity is high)				
Off body (unknown activity, likely showering / bathing)	0.25	0.39	0.33	0.33
Lying Down Sleeping	0.25	0.67	*0.00*	0.32
Lying Down Awake (resting)	0.41	0.46	*0.00*	0.46
Awake Non-Ambulatory (sitting / standing still)	**0.96**	*0.00*	0.70	*0.00*
Awake Intermittent Ambulation (walking, lower cadence)	*0.00*	*0.00*	0.25	**1.00**
Awake Purposeful Ambulation (walking, higher cadence)	*0.10*	0.25	0.37	0.54
**Bold:** High (80 to 90%) or Very High (>90%) probability. *Italics:* Low (10 to 20%) or Very Low (<10%) probability				
